# Rhytidoplasty Through Minimal Incisions (Rhythmic)

**DOI:** 10.1093/asjof/ojac059

**Published:** 2022-07-08

**Authors:** Porfirio Castillo-Campos

## Abstract

**Background:**

In the present report, an innovative alternative approach for rhytidoplasty is described. Rhytidoplasty Through Minimal Incisions (Rhythmic) is an effective, efficient, safe, and reproducible alternative procedure for facial rejuvenation. It has several advantages over traditional procedures.

**Objective:**

The main objective of this report is to describe the surgical technique of Rhythmic.

**Methods:**

The surgical technique for the foreheadplasty requires only a transverse ~7-mm incision ~1 cm behind the hairline at the midline. The upper and lower blepharoplasty are made with incisions of ~3 mm, and no suture is needed. Liposuction of the neck can also be performed, and the face-lift can be achieved through preauricular incisions.

**Results:**

Here, we summarize the results obtained in 741 patients subjected to rhytidoplasty through minimal incisions. The follow-up of these patients extended up to 20 years. This technique drastically reduced the length of scars; the eyebrows elevated 8-13 mm, and in some cases, the tip of the nose was also elevated ~7 mm. It minimized or eliminated the vertical frown lines and the horizontal furrows in the forehead. The use of this technique allowed an excellent definition of the cervicofacial angle in the patients.

**Conclusions:**

In conclusion, rhytidoplasty through minimal incisions drastically reduced the length of scars, bleeding, surgical time, avoided the use of drains, reduced costs, and shorted the convalescent period. It also offered the patients a natural appearance with minor complications.

**Level of Evidence: 2:**

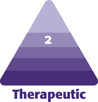

Plastic surgeons have described many procedures to improve the forehead and elevate the eyebrows.^1^ One of the most frequently used techniques consists of a complete coronal incision in combination with an extensive face-lift.^2^ In this procedure, depending on the height of the forehead and configuration of the hairline, the incision is made anterior or posterior to the hairline. The coronal incision has based its effect on the traction, and some authors have mentioned the relation between the resection of the skin with the elevation of the eyebrows.^3^ The technique emphasizes the skin traction and the deep stitches of suspension to elevate structures to maintain them in the desired position. However, excessive tension on the sutures is followed by circulatory impairment of the flap and subsequent alopecia or skin necrosis.^4^ Furthermore, the coronal incision causes scalp dysesthesias and scalp or prefrontal hairline scarring and the potential injury to the frontal branch of the facial nerve, and excessive elevation of the frontal hairline.^4^

An alternative endoscopic approach has been developed with small incisions and without skin resection.^5-8^ In endoscopic browlifting, 3-5 transverse or vertically oriented incisions of 1 cm are made 1 cm above the hairline. To avoid early brow ptosis, different methods of fixation of the soft tissue to the bone have been used, including titanium miniplates, 2-0 nylon sutures, absorbable screws, and cortical tunnels. However, this method is challenging to learn and requires repeated hands-on cadaver practice, a gradual introduction into clinical practice, and expensive and sophisticated equipment. Therefore, the endoscopic approach increases the cost and surgery time and, often, does not provide adequate exposure for visualization and preservation of the nerves that penetrate the muscle.^9^

In the cases of blepharoplasty and face-lift, different techniques have been described.^10,11^ In the transconjunctival approach for the removal of herniated fat, it has been reported that there was no need for skin resection in the lower eyelid.^12^ Likewise, a different technique was described that consisted of returning the herniated fat to the orbital cavity and retaining it by continuous sutures of the capsulopalpebral fascia either to the dehiscent portion of the orbital septum or to the periosteum of the lower orbital rim.^13,14^ It is worth mentioning the lower lid blepharoplasty with lateral retinacular and orbicularis suspension technique which also at the time challenged traditional approaches as extensive dissection of the orbicularis or skin and muscle flaps performed in lower blepharoplasty leads to denervation of the muscle and does not result in improved results. This technique has the advantage of improving the appearance of the eyelid-cheek junction and improving the lower eyelid’s appearance.^15^ For face-lift, different incisions in front of the ear have been performed. Likewise, it has been proposed to undermine the skin in the subcutaneous plane and reposition the deep tissue, where the skin and the platysma muscle are left together and raised as a single unit.^16,17^ Subsequently, different techniques have been described that are variations of the basic subcutaneous technique or the sub-SMAS (superficial musculoaponeurotic system) technique.^17-19^

The main objective of the current report is to describe an alternative approach for rhytidoplasty. This innovative surgical technique is rapid, easy, reproducible, requires minimal incisions, and is performed without endoscopy. The acronym Rhythmic has been coined to term it, which stands for *Rhy*tidoplasty *Th*rough *Mi*nimal *I*ncisions, *C*astillo-*C*ampos technique. Rhythmic offers enormous advantages for the patients, such as the elimination of the coronal, subcilial, and postauricular incisions, obtaining excellent jawline and cervicofacial angle definition, and no elevation of the hairline. Rhythmic is ideal for male patients with baldness and those who have previously undergone second or third traditional rhytidectomy.

## METHODS

Briefly, the continuity of the frontalis muscle with the occipitalis muscle through the galea aponeurotica was extensively studied in 7 cadaver dissections. Furthermore, to demonstrate this functional continuity, 12 electromyographic studies were done, in individuals ranging from 25 to 50 years old, allowing us to determine action potentials in both muscles, as well as the other muscles including the corrugator supercilli, the most important depressor of the brow. The electromyography (EMG) procedure included implanting electrodes in each muscle group to measure the force of contraction (right and left frontal muscle belly, right and left corrugator, pyramidal muscle, and occipital muscle). The electrodes were connected to the electromyograph, and individual recordings were obtained for each muscle group.

The only instruments used are a completely insulated, single-skin hook,^20,21^ with the ends free of insulation, and a straight blunt point semi-malleable elevator with a 20° curvature at its end. Both instruments have been designed explicitly for Rhythmic by the author.

The author confirms that all ethical guidelines in the Declaration of Helsinki were followed. In addition, written consent was provided, by which the patients agreed to the use and analysis of their data.

### Surgical Technique

#### Foreheadplasty Through a Minimal Incision

**Figure 1.**
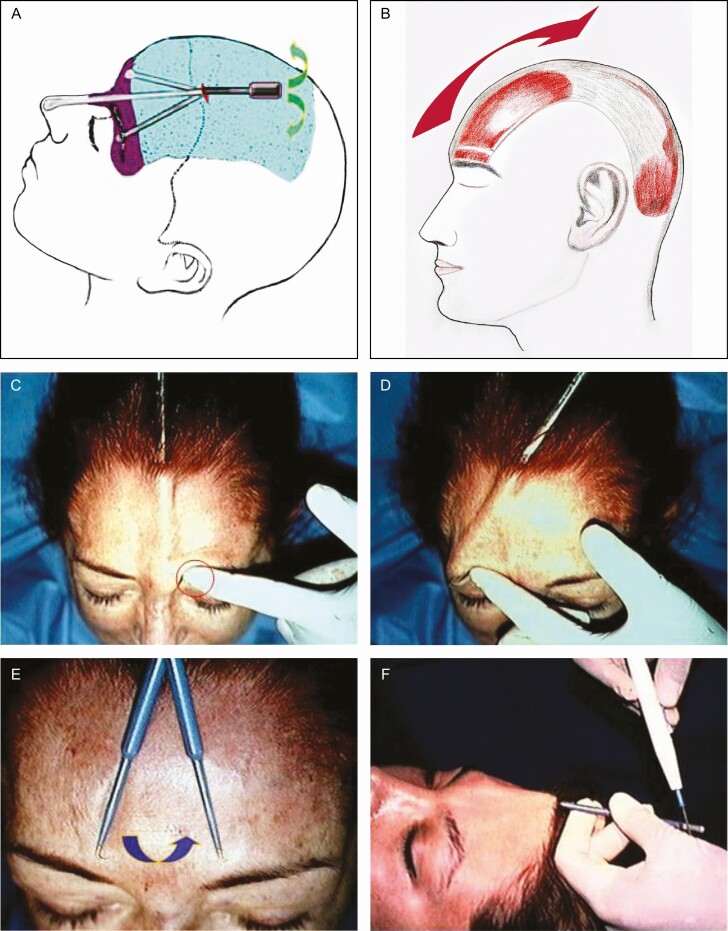
Diagram describing the 2 planes of dissection in the foreheadplasty with (A) a minimal incision and (B) the “elastic band” effect created by releasing of the frontalis muscle. (C, D) Preservation of the supraorbital and supratrochlear nerves. When placed in contact with an electrocautery, (E) a skin hook is employed to sever the corrugator and procerus as well as (F) the frontalis muscle fibers.

**Figure 2.**
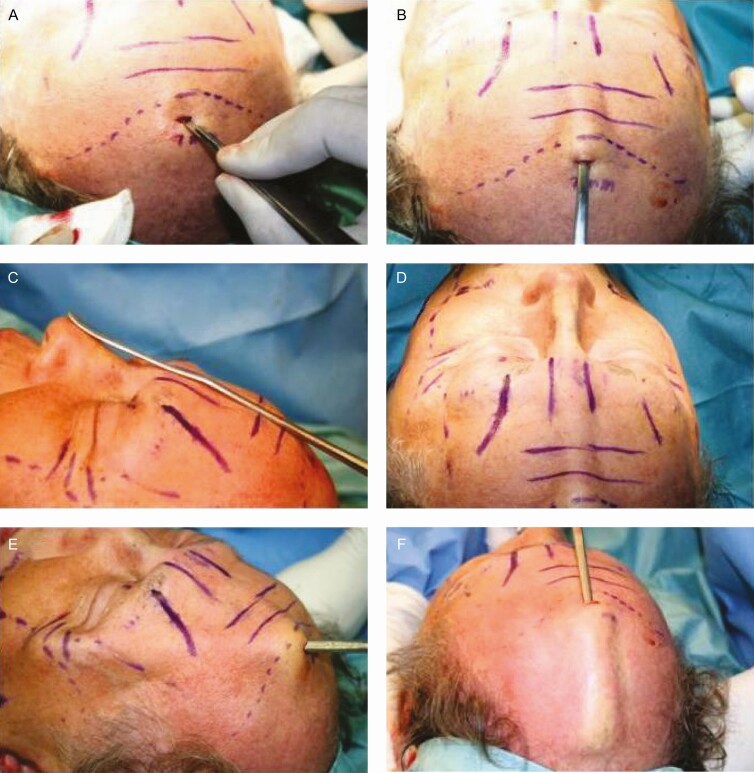
(A, B) Preoperative marking behind the hairline (dashed line) at the midline indicates the 7-mm incision. The dissection, using (C) a straight semi-malleable elevator, extends over (D) the dorsum of the nose and includes (E, F) the lateral orbital rim and posterior parietal areas.

To perform this procedure, only local anesthesia and intravenous sedation are needed. In Figures 1 and 2, the surgical technique is shown. It requires an initial transverse 7-mm incision, 1 cm behind the hairline at the midline. Figures 1A-D, 2A and B show the blind subgaleal blunt dissection in the frontal region. This dissection is performed in the whole forehead, and it is only subperiosteal 1 cm above the superior orbital rim to release the periosteum, and thus have the possibility of repositioning the eyebrows. The frontalis muscle takes origin from that galea over the superior frontal bone area and inserts into the superior orbicularis oculi muscle fibers. In the Rhythmic technique, the fused galea and periosteum layers are released above the supraorbital rim level. The galea must be released from the bone along the superior temporal line. In addition, a wide release of the cephalad by traction force of occipitalis muscle contraction.

It is important to recall that the subgaleal and subperiosteal plane is avascular; therefore, there is no bleeding. In addition, it should be mentioned that the sentinel vein is located 5-10 mm lateral and slightly below the frontozygomatic junction. This vein is an essential reference to locate the temporal branch of the facial nerve, which is located medially with respect to it. Over the superolateral angle of the orbital rim, the galea protects the frontotemporal branch of the facial nerve, and there are no subgaleal adhesions in this area.^22^ Therefore, the damage to the branch is unlikely because, as previously mentioned, the dissection plane is subperiosteal in the upper orbital rim of the orbital rim. Likewise, it should be mentioned that dense tissue within the galea temporal region generally requires to be approached with caution, avoiding electrocautery and using sharp dissection. Therefore, to protect the frontotemporal branch of the facial nerve, in the Rhythmic technique, the dissection is blunt, and the lateral portions of the frontal muscle are not weakened, so that patients can raise their eyebrows.

Figure 1C and D show that as with other methods where the anatomical landmarks are important, surface anatomic landmarks and digital palpation can safely protect the main branches of the supraorbital and supratrochlear nerves during Rhythmic^10,22,23^; however, care should be taken with the dissection as it is done blindly. It is worth mentioning that the main trunks of the nerves and their divisions run in the cephalic direction in the forehead, and they become more superficial, which allows the performance of myotomies without the risk of anesthesia in the region. This maneuver also limits the dissection of the brow’s head, avoiding its upward and lateral displacement.

It should be mentioned that a complication of the foreheadplasty is a severe elevation of the eyebrows. However, this is more related to the endoscopic procedures, where the superior orbital rim is generally dissected in its entirety, preserving the emergence of the supraorbital and supratrochlear nerves. However, this does not prevent the mobilization of the nerves in the cephalic direction where the flap moves, leaving a semicircular eyebrow arch look, which is not present in Rhythmic.

Figures 1D and 2C show that for the dissection of the forehead, a straight semi-malleable 20-cm long blunt point elevator with a curvature of 20° at its distal portion is employed. In Figure 2E, it can be seen that over the lateral orbital rim, the preperiosteal tissue needs to be released from its attachment to the superficial temporal fascia plane under the orbicularis muscle. This maneuver produces elevation of the brows and helps reduce the crow’s feet because of the cephalad transposition of the forehead flap. Figure 2D shows that the dissection extends over the nose’s dorsum, toward the nasal tip, achieving an elevation of approximately 7 mm. Furthermore, Figures 1A and 2F show that the dissection includes posterior parietal areas.

Figure 1E shows that the isolated single skin hook is inserted, the corrugator and procerus muscles are held, and the free end is used as a conductor when placed in contact with electrocautery in the coagulate mode to debilitate or severe these fibers. Figure 1F shows that debilitating on the frontalis begins 1 cm above the eyebrows extends to the hairline, leaving intact the lateral portions of the muscle, and it is done in a vertical direction with the dorsum of the hook’s curved end. Figure 1F shows that the weakening of the frontal muscle is carried out in the central region, at the limit of the middle and lateral third of both eyebrows, in order to reduce the transverse furrows of the forehead but leaving the lateral portions of the muscle intact so that the patient can continue raising the eyebrows without marking the transverse furrows before surgery. Aspiration of the air in the undermining area is then performed, and the small incision is sutured with only one stitch. To maintain the elevation of the eyebrows, compression and cephalic traction to the forehead are applied for 2 weeks with an elastic bandage. It should be noted that dyskinetic movements in the forehead might occur if the muscle-weakening is not performed uniformly. When the Rhythmic procedure is performed, the first muscles subjected to weakening are the corrugators. Once this is done, there is a decrease in the thickness of the glabellar region and at the level of the corrugator muscles. Subsequently, the weakening of the frontalis muscle is made, keeping the same thickness in the glabellar region and avoiding irregularities. It should be mentioned that many patients do not have furrows in the frontal and/or glabellar region, and consequently, it is not necessary to perform any muscle weakening.

#### Blepharoplasty Through Minimal Incisions

##### Upper *Blepharoplasty*: 

**Figure 3.**
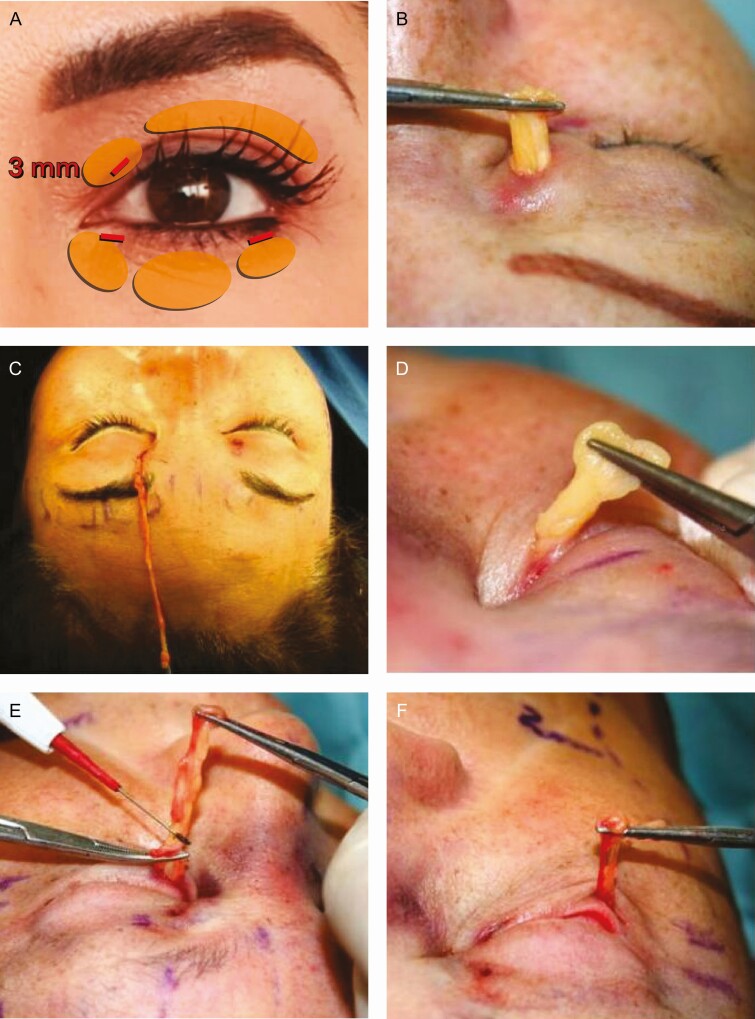
Blepharoplasty through minimal incisions. (A) Diagram showing the location of the three (3-mm) incisions used in Rhythmic. Resection of the (B) upper medial and (C) lateral fat compartments. Resection of the (D) medial, (E) central, and (F) lateral fat compartments in the lower blepharoplasty.

For the upper lid, a 3-mm incision is made at the level of the pretarsal crease line over the medial fat compartment as shown in Figure 3A. Likewise, Figure 3B and C show that the medial and the lateral fat compartment are removed, in most cases without skin resection for 2 reasons: (1) when the brows are elevated, the skin is redistributed, and (2) when the fat pads are removed the skin is contracted.

##### Lower *Blepharoplasty*

: In Figure 3A, it can be seen that a 3-mm incision is made 1 mm below the lash line laterally to the punctum. The orbicularis oculi muscle fibers are separated, and the orbital septum is pierced with fine Stevens’ scissors. Figure 3D-F show that by applying pressure to the ocular globe, the orbital fat is easily exteriorized and resected. Both the medial and the central fat compartments can be reached easily through this incision. For the lateral fat compartment, a 3-mm incision is made, 1 mm below the lash line near the lateral canthus, as shown in Figure 3F. Also, Figure 3E shows that the appropriate amount of the fat is removed, and the bleeding vessels are coagulated. It should be mentioned that there is no long-term deflation of the orbits with the technique described because only the fat that is protruding is extracted, not to have the effect of a “cadaveric orbit.” No sutures are needed.

#### Face-lift

Regarding the cheeks and neck, these areas were infiltrated with a local anesthetic and a vasoconstrictor (a mixture of 0.5% lidocaine with 1/200,000 epinephrine in cold physiological solution) after intravenous sedation. Liposuction of the neck is then performed. After this, a preauricular incision is made, extending from the tip of the earlobe to about 1.5 cm above the superior helix with a 45° angle, without any posterior auricular incision, except for a small one 5-mm incision surrounding the back portion of the earlobe. The anterior skin flap from the earlobe and above the superior helix is elevated in the superficial face-lift plane about 2 cm from the angle of the mouth. The dissection extends to the neck area. Thickening of platysma bands is corrected by strong pulling on the lateral part of the platysma muscle,^21^ and severing the bands by using a Colorado curved tip in the coagulation mode. Severing the lateral orbital portion of the orbicularis oculi muscle using a Colorado tip in coagulation mode significantly improves the crow’s feet.^24^

The SMAS plication suture is inserted 2 cm in front of the tragus at the level of the zygomatic arch. Then, using the same suture, a small portion of the SMAS is taken, and 1 or 2 plications aiming vertically toward the angle of the neck are performed. In some cases, one more placation toward the angle of the mouth is needed.

**Figure 4.**
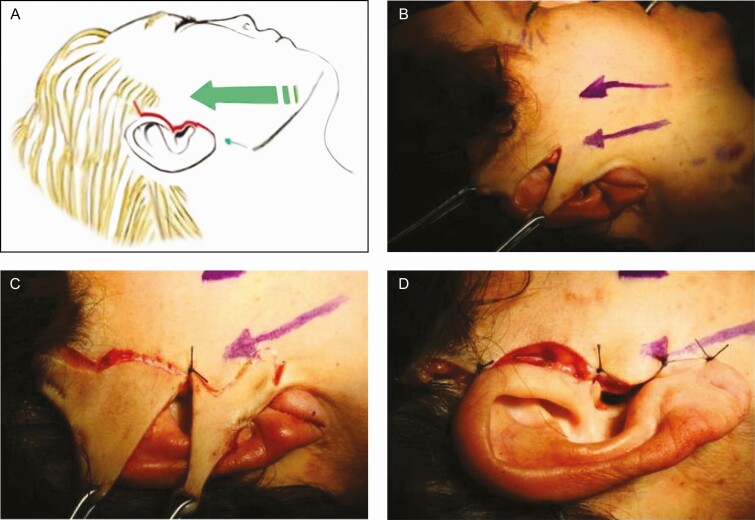
(A) Diagram showing the preauricular incision (red line) and the vector direction (green arrows) in face-lift through minimal incisions. The blue lines represent the undermining area. (B) The skin and subcutaneous vectors are going in a superolateral and vertical direction, respectively, and (C) a cut in the original skin triangle is made so that 2 new triangles are formed. Though the sideburn seems to show an over-elevation, this is due to the traction applied during the resection of the redundant skin. It is worth noting that the upper triangle does not contain hair follicles; therefore, the sideburn’s shape and position are not altered. (D) Lastly, the skin resection of the superior triangle is done following the shape of the superior helix.

Another plication parallel to the mandibular rim is inserted 3 cm below the mandibular angle in the platysma muscle through a preauricular incision. After subcutaneous dissection and SMAS and platysma plications, the skin for resection is in a triangle shape. Figure 4A shows that the direction of the skin traction should be 45°. The skin and subcutaneous vectors are going in a superolateral direction, and a cut in the original skin triangle is made in such a way that 2 new triangles are formed, as shown in Figure 4B and C. The first key stitch is placed just above the tragus to secure traction. Figure 4C shows that the skin resection of the superior triangle is done following the shape of the superior helix, with 45° angle at the superior end, to avoid displacement of the sideburn, while the resection of the inferior triangle is done following the posterior margin of the tragus leaving a small portion of remnant skin to avoid distortion of this anatomical structure after healing as shown in Figure 4D. The complete procedure is shown in the Video.

## RESULTS

In total, 741 consecutive patients between 22 and 80 years old from private practice underwent Rhythmic procedure. Forehead lift was performed on individuals below 40 years old, and the full face-lift on patients of 40 years and above (ratio of female vs male patients was approximately 9:1). The follow-up of these patients extended up to 20 years. The average age of the patients was as follows: in men, the range was from 49 to 80 years, with an average of 62.5 ± 7 years, while in women, the range was between 22 and 80 years, with an average of 57.3 ± 9.4 years. The youngest patient in the series, a 22-year-old female, underwent blepharoplasty only.

The percentage of each of the procedures performed was as follows: face-lift: 513 patients (69.2%), blepharoplasty 85 (11.5%), and foreheadplasty 143 (19.3%). The relationship with the main comorbidities is described in the was the following. Systemic arterial hypertension 136 patients (18.3%), diabetes mellitus 93 (12.6%), obesity 151 (20.4%), liver disease 20 (2.7%), gastritis-ulcer 16 (2.1%). The average follow-up time of the patients was of 1 year, ranging from 2 months to 20 years. However, in 2 patients, the follow-up extended for up to 10 years, and in 2 patients, it was up to 20 years.

The electromyographic studies in the frontalis and the occipitalis muscles showed potentials from 5.7 to 29.4 mVsec for the frontalis. In the case of the corrugators, the range of potentials in maximum contraction was 11.3-26.4 mVsec, with a significant predominance on the right corrugator was found. Unexpectedly, the other depressor muscles did not register any potential at all. Likewise, when the frontalis contraction was requested, in all cases, the occipitalis muscle registered potentials of 4.8 mVsec in average, demonstrating the physiological continuity of the occipitofrontalis musculoaponeurotic system (Table).

**Table.** Electromyographic Studies of Frontalis, Corrugators, and Occipitalis Muscles

Patient	Age (years)	Clinical evaluation	Electromyography (mVsec)							
		Wrinkles	R.F.	L.F.	F	R.C.	L.C.	C	OCC.	Group
1	37	Severe horizontal wrinkles lines	24.1	34.7	29.4	16.3	14.3	15.3	4.8	A
2	43	Severe flabellar frown lines	21.1	18.2	19.6	5.6	12.0	8.8	3.7	A
3	48	Moderated horizontal wrinkles lines	26.3	25.1	25.7	16.1	7.7	15.7	4.6	A
4	35	Moderated horizontal wrinkles lines	25.4	23.2	24.3	16.2	9.8	13.0	4.8	A
5	55	Light glabellar frown lines	5.1	6.3	5.7	18.5	4.1	11.3	4.5	B
6	50	Severe glabellar frown lines	16.4	12.3	14.3	28.6	24.3	26.4	2.8	B
7	30	Severe glabellar frown lines	7.3	8.5	7.9	20.6	6.4	13.5	4.4	B
8	32	Absence	20.5	15.0	17.7	16.9	15.2	16.5	3.6	C
9	50	Light horizontal wrinkle lines	22.6	20.9	21.7	28.9	23.2	26.0	2.9	C
10	25	Absence	11.0	10.2	10.6	16.3	9.8	13.0	4.1	C
11	34	Light glabellar frown lines	14.9	25.2	20.0	5.5	30.8	18.1	9.8	C
12	34	Absence	22.5	17.0	19.7	18.8	17.3	17.6	8.8	C

Group A includes patients in which the frontalis muscle dominated and horizontal wrinkles prevailed. On the other hand, group B consisted of patients in which corrugator muscles dominated and vertical furrows were persistently observed. In group C where apparently both muscle types exerted the same strength, horizontal wrinkles and vertical furrows could or could not be observed. C, average; F, average; LC, left corrugator; LF, left frontalis; RC, right corrugator; RF, right frontalis; mVsec, millivolts per second.

The clinical correlation of EMG studies in the frontalis and the occipitalis muscles is based on the greater strength of muscle contraction, the more and deeper furrows there are. In the case of the occipital muscle, when a strong contraction of the frontalis muscle occurs (when the patient is asked to raise the eyebrows), a contraction is recorded in the occipital muscle, which reveals a physiological continuity of the fronto-occipital musculoaponeurotic system and further confirms the concept of the elastic band, which is detailed in the Discussion.

**Figure 5.**
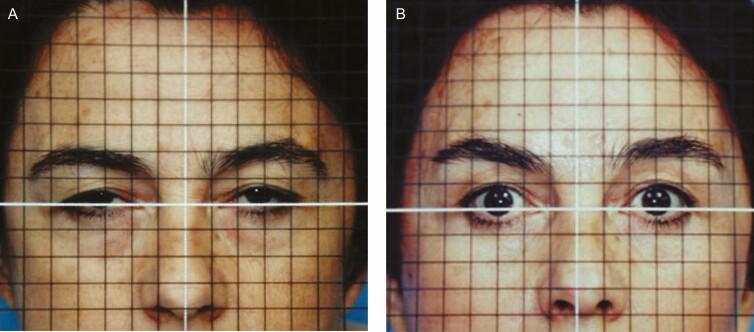
(A) Preoperative and (B) 6-month postoperative frontal views of a 50-year-old female patient subjected to foreheadplasty with a minimal incision showing eyebrows, upper eyelid margins, and nose tip elevation. It is worth emphasizing that no removal of fat and skin of the upper eyelid was performed. Interline space = 1 cm. Panel (B) shows a postoperative view 6 months after surgery. The patient’s follow-up was extended for a period of 10 years.

**Figure 6.**
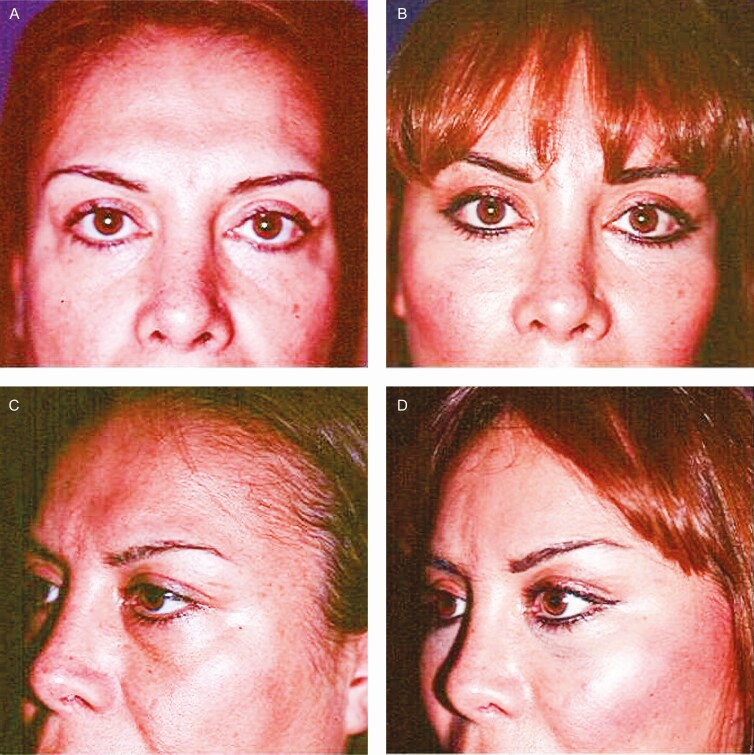
Preoperative (A) frontal and (C) left oblique projections. (B, D) Postoperative views of a 45-year-old female patient 6 months after surgery with minimal incisions. The tail of the brow is elevated (without skin resection in the upper eyelid). The patient’s follow-up was extended for a period of 1 year.

**Figure 7.**
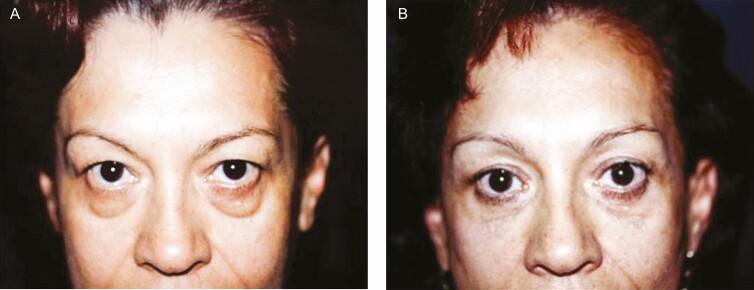
(A) Preoperative and (B) 6-month postoperative frontal views of a 65-year-old female patient subjected to upper and lower blepharoplasty through minimal incisions. The patient was also subjected to forehaeadplasty with a minimal incision. The patient’s follow-up was extended for a period of 20 years.

The outcome of 741 patients who underwent Rhythmic showed that this technique reduces the length of scars by avoiding 28 or more inches of incisions, as will be described further in the Discussion. Figures 5 and 6 show that the eyebrows are elevated from 8 to 13 mm; in some cases, an elevation of 5-7 mm approximately can also be obtained in the tip of the nose and in the upper eyelid margin (Rhythmic can also reduce or eliminate the vertical frown lines and horizontal furrows in the forehead as shown in Figure 7.

**Figure 8.**
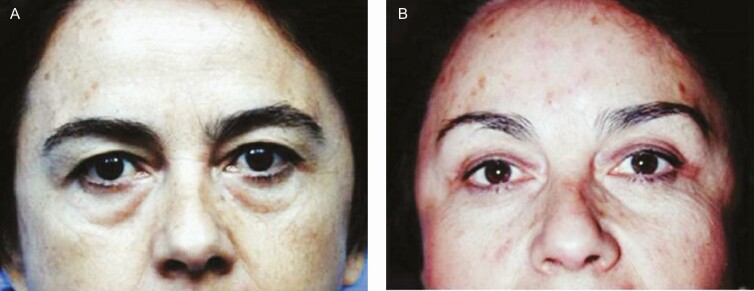
(A) Preoperative and (B) postoperative frontal views of a 50-year-old female patient, 10 years after surgery of the same patient shown in Figure 5. The patient’s follow-up was extended for a period of 10 years.

**Figure 9.**
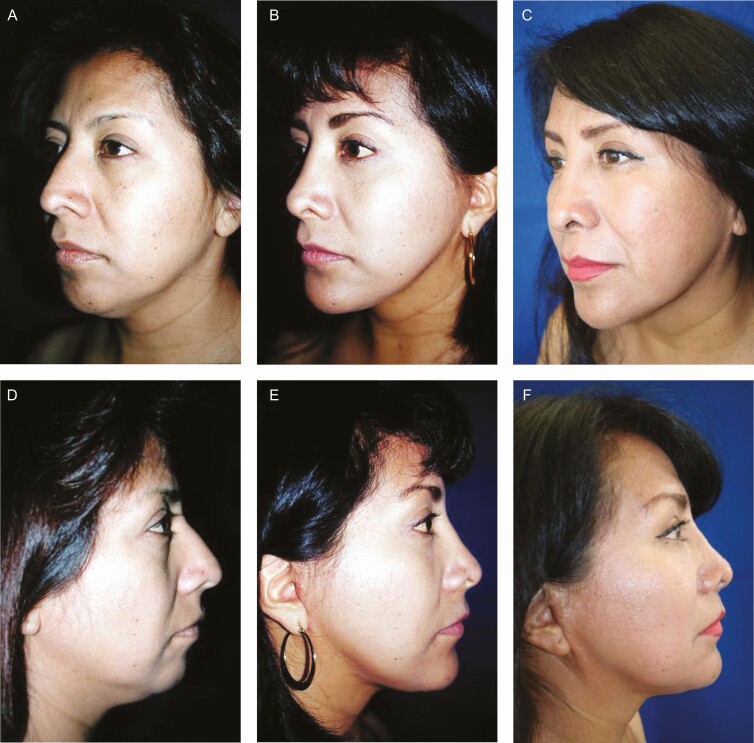
(A, D) Preoperative, (B, E) 6-month, and (C, F) 20-year postoperative lateral views of a 35-year-old female patient subjected to face-lift through minimal incisions. The patient exhibited a poor chin projection associated with an obtuse cervico-mandibular angle. Therefore, the procedure included the correction of the double chin and chin augmentation as well as rhinoplasty. Note that the sideburn is not displaced, and the preauricular incision is imperceptible. Likewise, the patient shows a (F) preauricular fold secondary to weight gain from Cushing’s syndrome after steroid treatment. It is worth mentioning that the image in panel F was taken 20 years after surgery, while the (E) follow-up image at 6 months does not show the fold since then the patient had not received steroid treatment.

**Figure 10.**
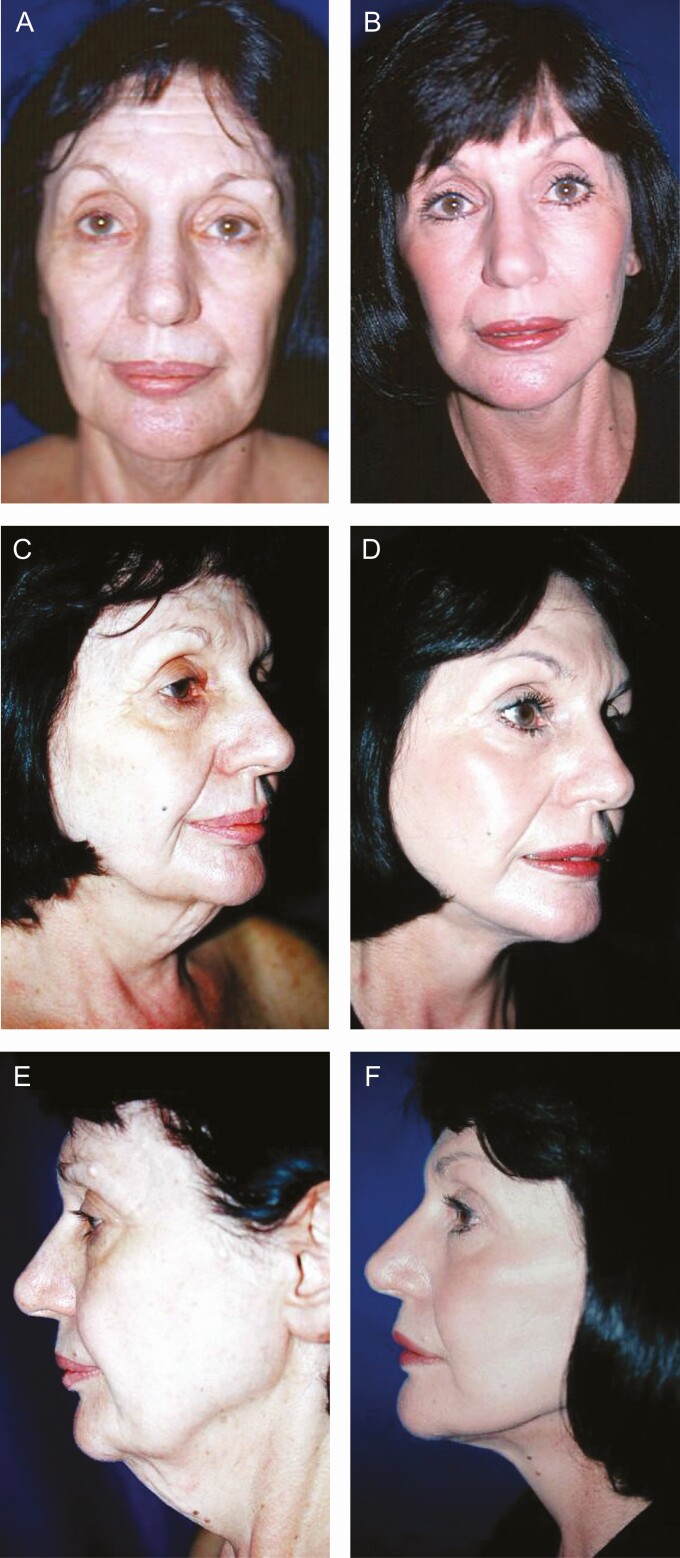
(A, C, E) Preoperative and (B, D, F) 6-month postoperative views of a 67-year-old female patient subjected to foreheadplasty, blepharoplasty, and face-lift through minimal incisions. The patient’s follow-up was extended for a period of 6 months.

**Figure 11.**
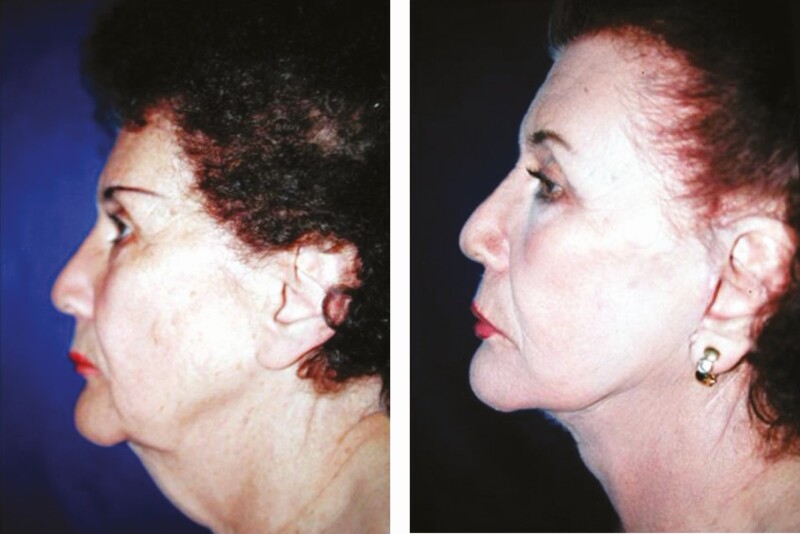
(A) Preoperative and (B) 6-month postoperative lateral views of a 71-year-old female patient subjected to face-lift through minimal incisions. The patient’s follow-up was extended for a period of 12 months.

**Figure 12.**
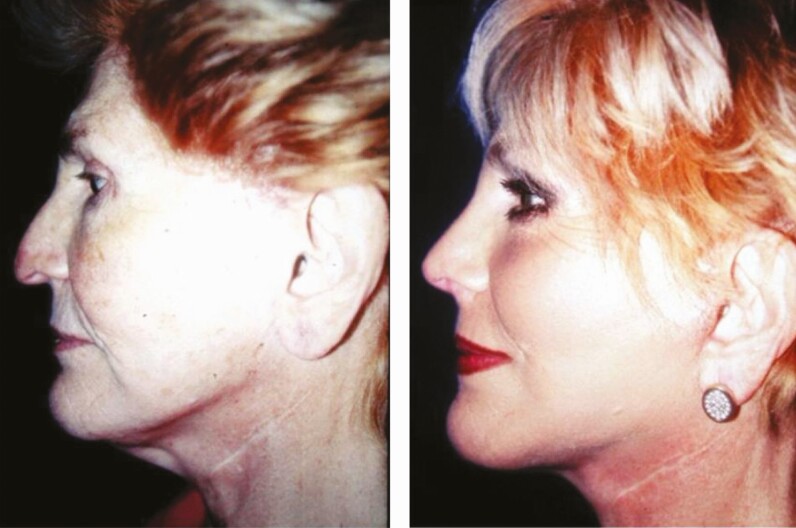
(A) Preoperative and (B) postoperative lateral views of a 65-year-old female patient 6 months following face-lift through minimal incisions and rhinoplasty. Neither blepharoplasty nor crow’s feet correction was performed. The patient had been subjected to 3 traditional rhytidectomies, 4 rhinoplasties, and neck surgery to correct cervical disk herniation before Rhythmic. Note the excellent definition of the cervicofacial angle and mandibular rim and a better projection of the malar region. The patient’s follow-up was extended for a period of 2 years.

Figures 8 and 9 show that regarding the upper and lower lids, the incisions are invisible with a natural look without any deformities. Furthermore, in Figures 10-12, it can be seen that a youthful face-lift appearance is achieved due to the definition of the cervicofacial angle, mandibular rim, and improved projection of the malar region.

Figure 3 shows that the Rhythmic technique allows obtaining the projected results without skin resection in the lids. Superior repositioning of the eyebrows corrects the descent that occurs with aging and gives an improvement to the hooding and redundancy of upper eyelid skin that was inappropriately treated with conventional blepharoplasty in the past, as can be seen in Figure 5. Likewise, as shown in Figures 5, 6, and 8, in approximately 97% of the cases using the Rhythmic technique, skin resection of the upper eyelids was not necessary, and in only 3% of the patients, it was necessary to perform skin resection. When the eyebrows are lifted, the skin of the upper eyelid is redistributed.

Likewise, Figures 5, 7, and 8 show that in contrast to the traditional blepharoplasty that involves the excision of both lax skin and muscle as well as excessive removal of fat, leaving some patients with a hollow orbit and a harsh operated appearance that accelerates the aging process, in the Rhythmic technique when the fat pads are resected, the skin gets retracted, restoring the patients a youthful appearance It is worth emphasizing that skin retraction that occurs in lower blepharoplasty can be observed in young patients as has been previously reported,^10,11^ and in elderly patients as can be observed in Figures 8 and 9 which is a novel finding of this study.

**Figure 13.**
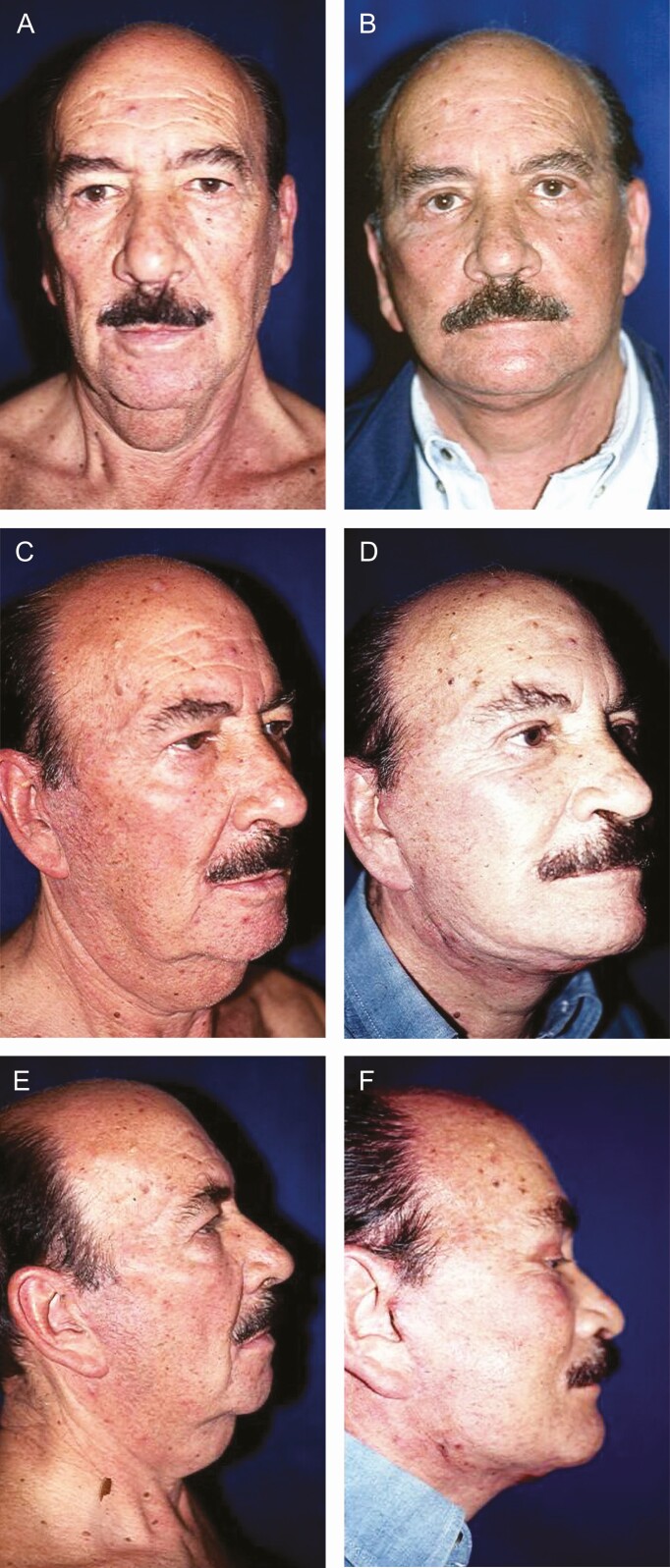
(A, C, E) Preoperative and (B, D, F) postoperative views of a 69-year-old male patient 6 months after face foreheadplasty through minimal incisions. The patient had undergone a traditional face-lift 2 years earlier. (E) The preauricular scar from the previous surgery. (F) After surgery with the Rhythmic technique, the scar is virtually imperceptible. The patient’s follow-up was extended for a period of 6 months.

Regarding the face-lift, the direction of the vector and the distance in which the force of traction is applied to the cheeks and neck, improve the final results in the face-lift using the Rhythmic technique given that a more vertical direction is used as can be seen in Figure 4, and a shorter distance between the preauricular incision and the middle line of the neck results in better traction and improved definition of the cervicofacial angle, shown in Figures 10-13. Furthermore, it is also known that the skin in the retroauricular region is always stretched as it is closely linked to the insertion of the sternocleidomastoid muscle, no matter the patient’s age, so it is unnecessary to perform an undermining of it. Though no patient required retroauricular incision that extended to the occipital region, in some patients (~0.5%), it was necessary to re-operate to correct the severe sagging of the cervical region. Lastly, ~3% of the patients presented temporary paresis due to postoperative inflammation of the buccal branch of the facial nerve, recovering entirely in all cases.

Likewise, concerning a possible elevation of the sideburn as a result of Rhythmic, Figure 4 shows that the ~1 cm 45° incision performed at the top of the helix, which helps to compensate flap elevation, also helps to prevent the elevation of the sideburn. For this reason, hair-bearing scalp rotation downward to the sideburn area and bringing the superior end of the preauricular incision anterior to the temporal hairline are unnecessary. The last maneuver may be helpful in Caucasian patients, but it does not work that well for patients with darker skin where the scar is more noticeable.

Lastly, complications associated with the Rhythmic technique are minor and infrequent. Actually, the risk of damage to the inferior oblique muscle is lower than in other techniques, and no patient has ever presented this complication. However, some patients (<2%) have referred transient diplopia in the immediate postoperative period because of edema and local anesthesia infiltration but never because of injury. In the total number of patients that underwent surgery, only a few cases of transient paresthesia were observed, and no facial nerve palsy was found. Furthermore, although hematoma due to bleeding from the superficial temporal vessels is a complication of face-lift surgery,^25,26^ in Rhythmic both acute and late hematomas are rarely seen (<1% of all cases), because meticulous hemostasis is performed (including superficial temporal vessels ligation) and compressive dressings are always applied during the first 24 hours after surgery. It should be mentioned also that there were no cases of infection or skin necrosis. Lastly, of all the cases subjected to foreheadplasty, a minimal fraction (approximately 1%) required some filler, and this happened at the beginning of the technique’s implementation.

In 2 cases, a small unilateral hematoma was developed in the left cheek, and another patient had a seroma in the forehead that required aspiration. These complications were found in patients who removed the dressing before time. Likewise, less than 1% of the patients referred frontal itching after surgery that disappeared a few days later. In only 2 cases, the itching lasted from 1 to 2 months. Some patients (<1%) had dimpling of the skin below the earlobe or glabellar depression. Earlobe dimpling was resolved spontaneously in 2-4 weeks on average, while glabellar dimpling or depression in some cases required lipoinjection.

## DISCUSSION

As described above, Rhythmic is a novel and innovative alternative for facial rejuvenation. This technique has many advantages over major rhytidectomy techniques in practice today, including the conventional rhytidectomy, deep-plane or composite techniques, limited incision approach (eg, lateral SMASectomy, S-lift, MACS-lift), and suspension suture lift (eg, APTOS, Contour Threads). Rhythmic significantly reduces the length of scars, the risk of complications, and helps patients to achieve a more natural look. Some of the results obtained with Rhythmic have been previously published in abstract form,^21,24,27-30^ and some parts of the technique have been discussed and reviewed by colleagues who have received training in Rhythmic directly from the author.^20,31,32^

Progressively fewer invasive techniques have been developed for facial rejuvenation. Whereas some of these techniques represent refinements of old methods for surgery of the cheeks and neck,^33-35^ Rhythmic represents an entirely new modality given that it involves the middle and lower thirds of the face, and the neck also implies a foreheadplasty and upper and lower blepharoplasty.

As an initial step in this study, EMGs were performed to demonstrate the anatomophysiological continuity of the fronto-occipital musculoaponeurotic system. This helps explain the elastic band effect that allows the traction of the frontal muscle with the consequent elevation of the eyebrows. In addition, with the data obtained, it was possible to identify the 3 groups of patients (Table). These studies helped in planning the surgical treatment. For example, in the group of patients classified as C, where there were no vertical furrows between the eyebrows or transverse forehead furrows, only the frontal area was dissected without muscle weakening.

One of the main features of Rhythmic is what the author called the “elastic band effect” shown in Figure 1B. The musculoaponeurotic system that performs mime for the upper third of the face comprises the subcutaneous fascia and the occipitofrontalis, corrugator supercilii, and procerus muscles. Understanding these muscles’ activity offers the possibility to correct horizontal furrows on the forehead, horizontal lines on the root of the nose, and glabellar frown lines. The forehead muscles and glabellar expression are superficially located, supplied by the facial nerve, and inserted into the skin. The occipitofrontalis muscle is a fibrous muscular sheet composed of 2 muscle bellies joined through an aponeurotic tissue that dresses the skull from the superior nuchal line to the supraorbital rims. This muscle is the only elevator of the brows and is responsible for the horizontal wrinkle lines of the forehead.^36^

As described in the Methods section, the physiological continuity of this system was demonstrated by measurements of action potentials. Therefore, an elastic band effect can be achieved by avoiding the antagonistic action of the depressor muscles (by releasing, debilitating, or severing them) and releasing the frontalis muscle from its insertion on the supraorbital rim, while the occipitalis muscle retains its insertion, exerting traction in the cephalic direction of the frontalis muscle, consequently producing the elevation of the eyebrows. On average, this maneuver results in an 8- to 13-mm elevation of the eyebrows. It is also worth mentioning that the foreheadplasty procedure was performed in 656 patients. It is also important to mention that an elastic dressing must be applied to the patient’s forehead for at least 2 weeks to avoid the action of gravity and prevent edema. In this way, the eyebrows will reattach at that higher level.

Measurements of action potentials also yielded interesting data on the functional actions of the forehead muscles. Previous studies have suggested that depression of the eyebrows seems to be attained by the orbicularis oculi and the corrugator muscles, and to a lesser degree, by the procerus muscle, drawing the eyebrows downward and medially. However, the results of the electromyographic studies indicated that only the corrugator supercilia muscles registered action potential after requesting to frown. This finding suggests that the orbicularis oculi and the procerus muscles may not participate in the depression of the eyebrows. In contrast, when the elevation of the eyebrows was requested, in all cases, the occipitalis muscle registered action potentials, indicating its participation in this event, providing evidence for the elastic band effect.

Superior repositioning of the eyebrows corrects the descent that occurs with aging and gives a drastic improvement to the hooding and redundancy of upper eyelid skin that was inappropriately treated with conventional blepharoplasty in the past. In the Rhythmic technique, in approximately 97% of the cases, skin resection of the upper eyelids was not necessary. Instead, when the eyebrows are lifted, the skin is redistributed. Likewise, in contrast to the traditional blepharoplasty, which involves the excision of both lax skin and muscle, and excessive removal of fat, that leaves patients with a hollow orbit and a harsh, operated impression, and an older appearance,^37^ in the Rhythmic technique when the fat pads are resected, the skin gets retracted, restoring the patient’s youthful appearance.

Regarding the resection of the fat pads, the results obtained with the Rhythmic technique are in agreement with a previous report by Parsa et al who published a comparative study between standard blepharoplasty in 1 lower eyelid and capsulopapebral fascia hernia repair in the other lower eyelid.^38^ The results of this study show that similar aesthetic outcomes can be obtained in treating palpebral bags with the 2 different techniques.

In addition, skin resection of the lower eyelids was not necessary in any case, avoiding some possible complications of the conventional blepharoplasty, such as scleral show or ectropion which result in a lack of natural expression, as well as the complications of the transconjunctival approach, such as scaring that causes a foreign body sensation, entropion, and diplopia.^39^

In the cheeks and the neck, only a preauricular incision is necessary, achieving a better definition of the cervicofacial angle due to the superolateral direction of SMAS and skin traction, in comparison to the conventional technique in which the soft tissues are pulled in a transversal and not in a vertical direction which is the manner in that the tissue descends naturally. Through the SMAS plications, Rhythmic achieves volumetric resculpture of the malar region following J. W. Little’s philosophy.^40,41^ As previously reported,^42^ the author shares the disappointment about the poor long-term results of the anterior corset platysmaplasty. For this reason, the bands of the platysma muscle in Rhythmic are corrected by severing them according to what was detailed in the surgical technique section.

The Rhythmic technique allows favorable results in both male and female patients with neck flaccidity because the traction vector at the skin and SMAS level is in the vertical direction, in such a way that all the redundant skin of the cheeks and neck can be resected at the preauricular incision. Likewise, the reason why better results are obtained in the neck using the preauricular incision is that the distance between the incision and the middle line of the neck is less in the Rhythmic technique causing larger skin traction. Furthermore, in the traditional technique, the skin from the neck is pulled in an oblique direction and not in the natural vertical direction. Likewise, another limitation of the conventional approach is imposed by the skin of the postauricular region that is firmly attached to the mastoid fascia and generally very difficult to dissect with the risk of damage to the great auricular nerve and the external jugular vein. Lastly, in order to obtain a good neck definition, an important traction of the postauricular flap is applied in the traditional technique, which causes great tension of the incisions resulting in poor quality scaring and skin necrosis of the region, which is particularly apparent in smokers.^43^ For these reasons, the postauricular incision is not performed in the Rhythmic technique.

Although there are few contraindications to the procedure, training is required for any new procedure. According to this, the surgeon’s learning curve could be a limitation for implementing the technique. However, once mastered, one of the multiple advantages of the technique is its significant reduction of surgical time. Hence, the total operating time for the complete procedure is 1.5-2 hours.

## CONCLUSIONS

The current report describes a novel method for rhytidoplasty through minimal incisions (also called Rhythmic). Rhythmic produces cephalic and back traction that elevates the eyebrows. This effect is because the release of the frontalis muscle produces relaxation, and given its anatomical and functional continuity with the occipitalis muscle that maintains its natural insertion and tension, this maneuver results in what the author called “elastic band effect.”

Rhythmic technique allows achieving excellent results without the need of skin resection in the lids. The upper and lower eyelid skin is retracted when the fat is removed from its compartments, which occurs in young and older patients.

The direction of the vector and the distance in which the force of traction is applied to the cheeks and neck improve the final results in the face-lift using the Rhythmic technique given that a more vertical direction is used and a shorter distance between the preauricular incision and the middle line of the neck results in better traction and improved definition of the cervicofacial angle.

In summary, Rhythmic is an effective, efficient, safe, and reproducible procedure that has the following advantages: (1) elimination of the coronal, subcilial, postauricular, and occipital incisions; (2) obtaining excellent mandibular rim and cervicofacial angle definition; (3) drains are not necessary; (4) endoscopy is not required; (5) no elevation of hairline; (6) ideal for patients with male pattern baldness; and (7) excellent results in patients who have previously undergone second or third conventional rhytidectomy.

**Figure 14.**
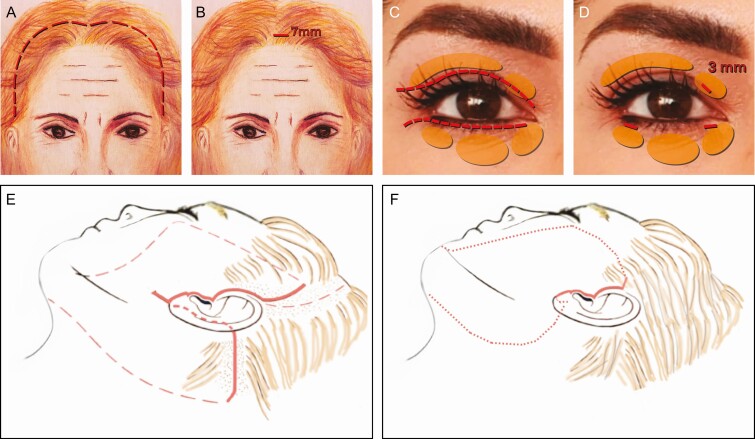
Diagram compares the incisions’ length between the traditional technique (left) and Rhythmic (right). (A) The upper panels compare the coronal incision (dashed red line) to the (B) minimal incision (7 mm) in Rhythmic. Panels C and D compare the incisions in the (C) traditional blepharoplasty (dashed red line) with (D) the minimal incisions (3 mm) in Rhythmic, one in the upper lid and two in the lower lid. Panels E and F compare the (E) pre- and postauricular incisions of the traditional technique with the (F) single preauricular incision in Rhythmic.

In addition, it is worth mentioning that Rhythmic eliminates an average of 28 inches (~70 cm) of scars, considering that on average, the length of the incisions in traditional rhytidectomy has the following dimensions depending on gender and head circumference: the coronal incision is approximately 10-12 inches and the 4 blepharoplasty incisions (upper and lower) total approximately 8 inches. In addition, the bilateral pre- and postauricular incisions sum approximately 10-12 inches. Figure 14 compares the Rhythmic technique with the traditional rhytidectomy in terms of length of incision. Lastly, the Rhythmic technique also reduces bleeding, surgical time, recovery time, materials, and equipment and produces a more natural and youthful appearance in the patients.
